# Trends in Sudden Death Among Schizophrenia Inpatients

**DOI:** 10.3390/medicina61122081

**Published:** 2025-11-22

**Authors:** Andreea-Violeta Popa, Petru Iulian Ifteni, Paula Simina Petric, Daniel Țâbian, Andreea Teodorescu

**Affiliations:** 1 Faculty of Medicine, Transilvania University of Brasov, 500036 Brasov, Romania; andreea.popa@unitbv.ro (A.-V.P.); daniel.tabian@unitbv.ro (D.Ț.);; 2 Clinical Hospital of Psychiatry and Neurology of Brașov, 500123 Brasov, Romania; 3 Forensic Medicine Service, Brasov County Clinical Emergency Hospital, 500326 Brasov, Romania

**Keywords:** schizophrenia, sudden death, autopsy, cardiovascular diseases, antipsychotic agents, inpatient mortality, forensic medicine, risk factors, cause of death, psychiatric hospitals

## Abstract

*Background and Objectives:* Schizophrenia is associated with a 15–20-year reduction in life expectancy, with cardiovascular disease as the leading cause. Sudden unexpected death is common in this population, often linked to structural heart disease, antipsychotic use, and overlapping cardiometabolic, autonomic, and drug-related factors. This study aimed to determine the incidence and causes of sudden unexpected death among schizophrenia inpatients between 2014 and 2024 and compare these findings with historical data from the same institution. *Materials and Methods*: We conducted a retrospective cohort study of schizophrenia inpatients admitted from January 2014 to December 2024. Hospital records were reviewed to identify sudden and unexpected deaths, verified by the Forensic Medicine Service Brașov. Sudden death was defined as death in an asymptomatic patient or within one hour of new symptom onset, excluding suicide, homicide, or accidental overdose. In accordance with Romanian legislation, an autopsy was performed in every case. *Results*: Over the 10-year period, six schizophrenia inpatients (mean age 53.2 ± 17.8 years) died suddenly. All had long-standing schizophrenia (mean illness duration 28.7 ± 17.7 years) and were receiving second-generation antipsychotics. Cardiovascular comorbidity was present in three cases. All patients received antipsychotic treatment within 24 h before death. No deaths occurred within the first 24 h of admission; one occurred within 48 h. Compared with the 1989–2013 cohort, which included 57 sudden deaths, the incidence during 2014–2024 declined substantially (0.27% vs. 0.79%). *Conclusions*: The incidence of sudden unexpected death among schizophrenia inpatients declined significantly over the past decade compared with the 1989–2013 cohort, reflecting improved multidisciplinary care, prompt transfer to general hospitals, and wider use of second-generation antipsychotics. Autopsy findings emphasize the continuing importance of cardiovascular disease and airway obstruction as preventable causes of sudden death in this population.

## 1. Introduction

Schizophrenia is associated with one of the highest risks of premature mortality among psychiatric disorders, with a 15–20 year life expectancy gap [1]. Mortality in schizophrenia has been increasingly recognized as a measurable clinical endpoint, with causes of death primarily involving cardiovascular disease, cancer, and suicide [2]. Within this context, sudden unexpected death—classically defined as an unexpected death occurring within approximately one hour of symptom onset [3]—has been a persistent and alarming phenomenon among psychiatric inpatients with schizophrenia. Sudden deaths in psychiatric hospitals have been documented for decades, but their causes remain poorly understood [4,5]. Earlier studies relying on death certificates found that some of these fatalities were classified as ‘unexplained,’ underscoring a historical gap in knowledge [6]. In a 10-year postmortem study, cardiovascular disease accounted for nearly a quarter of deaths in schizophrenia, while about 11% remained unexplained even after autopsy, pointing to the role of occult arrhythmogenic mechanisms [7]. Similarly, a larger forensic autopsy series of 391 schizophrenia patients reported cardiovascular disease as the cause in nearly 79% of natural deaths, with cardiac arrhythmias confirmed in a subset, and about 9.7% still classified as undetermined despite complete postmortem investigation [8].

Epidemiological data indicate that patients with schizophrenia face a dramatically elevated risk of sudden death compared to the general population. Large population-based analyses have shown that having a schizophrenic disorder confers one of the highest relative risks of sudden cardiac death (SCD) among all psychiatric conditions; for example, a recent nationwide study in Denmark found that patients with schizophrenia had an adjusted hazard of SCD approximately 4.5 times higher than peers without psychiatric illness [9]. Similarly, prospective registry data show that individuals with schizophrenia are overrepresented among young SCD cases by more than tenfold, with higher burdens of cardiovascular risk factors, unwitnessed arrests, prolonged time-to-discovery, and asystole—all factors associated with poor survival outcomes [10].

Multiple pathophysiological mechanisms have been implicated in the heightened vulnerability to sudden death among patients with schizophrenia. Cardiovascular comorbidity is a central factor: up to 40% of all schizophrenia patients die of cardiovascular causes (including coronary artery disease, myocardial infarction, myocarditis), reflecting the high prevalence of metabolic syndrome, smoking, and other risk factors in this group [11]. In addition, schizophrenia is linked to chronic low-grade inflammation and abnormal vascular endothelial function, with elevated levels of biomarkers such as VEGF, ICAM-1, and VCAM-1 indicating increased vascular stress and inflammation. These abnormalities are associated with higher blood pressure, insulin resistance, and overall cardiovascular risk, suggesting that endothelial dysfunction may play a key role in the vascular pathology of schizophrenia [12]. Patients also experience higher rates of arrhythmia, syncope, and heart failure than the general population, indicating underlying autonomic dysfunction [13]. This autonomic imbalance—typically manifesting as reduced heart rate variability and increased sympathetic tone—has been demonstrated in both acutely ill patients and their first-degree relatives, indicating a heritable component of cardiac dysregulation [14,15]. Genetic and molecular findings provide additional insight into this susceptibility. Recent Mendelian randomization studies further suggest a bidirectional relationship between psychiatric illness and cardiac pathology, with hypertrophic cardiomyopathy identified as a mild but significant risk factor for schizophrenia [16]. More recent genetic studies have also found shared genetic factors between schizophrenia and arrhythmic disorders, especially Brugada syndrome, and showed that schizophrenia itself may increase the risk of abnormal heart rhythms and higher heart rate [17]. A recent forensic-genomic study confirmed that most sudden deaths in schizophrenia are linked to structural heart disease or antipsychotic exposure, while only a minority remain unexplained, suggesting that rare inherited arrhythmia syndromes may account for some cases [18]. More recently, single-nucleus RNA sequencing has identified CACNA1A overexpression in cardiomyocytes as a potential biomarker for sudden unexplained death in schizophrenia, implying a role for subtle ion-channel dysregulation in these cases [19]. The study also found changes in other calcium-channel and contractile genes (such as CALN1, ANKRD1, and PLN), indicating broader cardiomyocyte remodelling that may contribute to sudden death in schizophrenia [19]. Beyond structural and genetic factors, dysregulation of cardiac ion channels and altered dopaminergic, serotonergic, and adrenergic receptor signalling—key targets of antipsychotic drugs—may further heighten arrhythmogenic vulnerability [20,21,22,23]. Additionally, evidence from molecular and translational studies indicates that oxidative stress dysregulation, mitochondrial dysfunction, and elevated inflammatory mediators such as IL-6 and TNF-α are prominent in schizophrenia, suggesting that systemic biological stress—potentially affecting cardiac tissue—may further contribute to sudden death vulnerability [24].

Metabolic disturbances such as psychogenic polydipsia with severe hyponatremia have been described as rare but important contributors to sudden death in schizophrenia, highlighting the importance of electrolyte monitoring and clinical vigilance [25]. Manu et al. (2011) [4] reported that unexplained sudden deaths in psychiatric patients were strongly associated with dyslipidaemia and diabetes as comorbidities, whereas the type of antipsychotic medication (first- vs. second-generation) did not fully account for the risk. Such findings implicate intrinsic patient factors—obesity, insulin resistance, and hypertension—in accelerating atherosclerotic cardiovascular disease, which in turn can precipitate fatal events.

Antipsychotic medications have been another focus of etiologic research, given their known cardiometabolic side effects [26]. Both first-generation (typical) and second-generation (atypical) antipsychotics can prolong cardiac repolarization (QT interval), increasing the risk of ventricular arrhythmias such as torsades de pointes [27,28]. Recent reviews highlight that antipsychotic cardiotoxicity involves not only QT prolongation but also direct myocardial injury, and suggest that newer agents or adjunct cardioprotective strategies may help reduce this risk [29,30]. Early pharmacoepidemiological evidence from a large U.S. cohort study by Hennessy et al. (2002) [31] showed that patients with treated schizophrenia had a 1.7–3.2-fold higher rate of cardiac arrest and ventricular arrhythmia compared with non-psychiatric controls, with risk particularly elevated at higher thioridazine doses, suggesting a dose-dependent relationship between antipsychotic exposure and arrhythmic events. A meta-analysis by Salvo et al. (2016) [32] confirmed that the risk of sudden cardiac or unexpected death varies across individual antipsychotics, with the highest odds observed for thioridazine, clozapine, risperidone, and haloperidol; this risk correlated with the degree of hERG potassium channel blockade. In line with this, Wu et al. (2015) conducted a nationwide case-crossover study in over 17,000 patients and found a 1.5-fold higher risk of ventricular arrhythmia or sudden cardiac death during antipsychotic exposure, especially with short-term use and agents showing high hERG-blocking potency such as haloperidol, thioridazine, and risperidone [33]. Moreover, Itoh et al. (2016) [34] demonstrated that nearly one-third of patients with acquired long QT syndrome carry mutations in congenital LQTS genes, suggesting that latent genetic variants may predispose to drug-induced arrhythmias. Supporting this, Esposito et al. (2025) [35] identified myocardial fibrosis and contraction band necrosis in young schizophrenia patients who died suddenly while on antipsychotic therapy, underscoring that antipsychotics can induce arrhythmogenic cardiac remodelling even in the absence of classic cardiovascular risk factors. Chen et al. (2022) [36] reported nine in-hospital SCD cases showing mainly ischemic or structural heart disease, with some unexplained deaths, emphasizing the need for continuous ECG monitoring during hospitalization.

Autopsy-based data from China suggest that persistent psychiatric episodes are an independent risk factor for sudden unexplained death (OR ≈ 4.2), while clozapine treatment appears protective, reinforcing the importance of maintaining psychiatric stability to reduce sudden mortality risk [37].

Recent nationwide data suggest that antipsychotic formulation may influence sudden cardiac death risk: compared with oral monotherapy, LAI monotherapy, and especially LAI–oral combinations were associated with higher SCD risk, particularly among patients with cardiovascular comorbidities [38]. These findings have generated discussion, including a recent letter to the editor [39] emphasizing that the results indicate association rather than causation, and highlighting the importance of considering diagnostic heterogeneity and illness severity when interpreting the data. Overall, the study underscores the value of cardiovascular assessment and careful monitoring when prescribing LAIs or combination regimens, without discouraging their use in clinically justified cases.

Scientometric analyses confirm that antipsychotic-induced sudden cardiac death remains a major research focus, with growing emphasis on understanding mechanisms, implementing safety monitoring, and refining risk–benefit assessment of newer agents such as clozapine and olanzapine [40].

Despite increasing recognition of these multifactorial mechanisms, few studies have systematically examined whether the incidence and aetiologies of sudden unexpected death in schizophrenia have changed under modern treatment and monitoring conditions. Addressing this gap is essential to evaluating real-world progress in patient safety. Accordingly, this study aimed to determine the incidence and causes of sudden unexpected death among schizophrenia inpatients between 2014 and 2024 and to compare these findings with historical data from the same institution (1989–2013).

## 2. Materials and Methods

### 2.1. Study Design and Setting

This was a retrospective observational cohort study conducted at the Clinical Hospital of Psychiatry and Neurology, Brașov, Romania. The hospital is an academic psychiatric centre with 100 beds dedicated to acute psychiatric care. The study aimed to identify the incidence and causes of sudden unexpected death among schizophrenia inpatients during a 10-year period (2014–2024). The methodology follows established principles for retrospective clinical research and hospital-based mortality studies in psychiatry.

### 2.2. Study Population

The study included all inpatients diagnosed with schizophrenia who died suddenly and unexpectedly during hospitalization. Cases were identified through a systematic review of hospital records, and all deaths were confirmed through the Forensic Medicine Service, Brașov.

### 2.3. Definition of Sudden Death

Sudden unexpected death was defined as a rapid, unforeseen fatal event occurring in an otherwise stable inpatient or within one hour of new symptom onset, consistent with definitions used in psychiatric settings [6,41]. Deaths due to suicide, homicide, trauma, or accidental overdose were excluded. Both natural causes (for example, cardiovascular or respiratory) and abrupt non-natural physiological mechanisms, such as mechanical asphyxia due to airway obstruction, were included when confirmed as sudden and unexpected by autopsy.

### 2.4. Data Collection and Ethics

Data extracted from hospital records and autopsy reports included demographic characteristics, illness duration, length of stay prior to death, medical comorbidities, psychopharmacological treatments administered within 24 h prior to death, and autopsy findings. In Romania, autopsy is legally mandated for all inpatient deaths in a psychiatric ward and was therefore performed in each case by board-certified pathologists at the Institute for Legal Medicine, Brașov.

The study was approved by the Ethics Committee of the Clinical Hospital of Psychiatry and Neurology Brașov (decision no. 13, 13 November 2023). As this is a retrospective study using anonymized data extracted from existing medical records, written informed consent was not required. All procedures complied with the Declaration of Helsinki and the European Union General Data Protection Regulation (GDPR).

### 2.5. Statistical Analyses

Descriptive statistics were calculated for demographic and clinical variables. Continuous variables are expressed as means with standard deviations, and categorical variables as absolute numbers and percentages. Comparisons with previously published data [6] were made using chi-square or Fisher’s exact test for categorical variables and Student’s *t*-test for continuous variables. A two-tailed *p*-value of <0.05 was considered statistically significant.

## 3. Results

**Figure 1:**
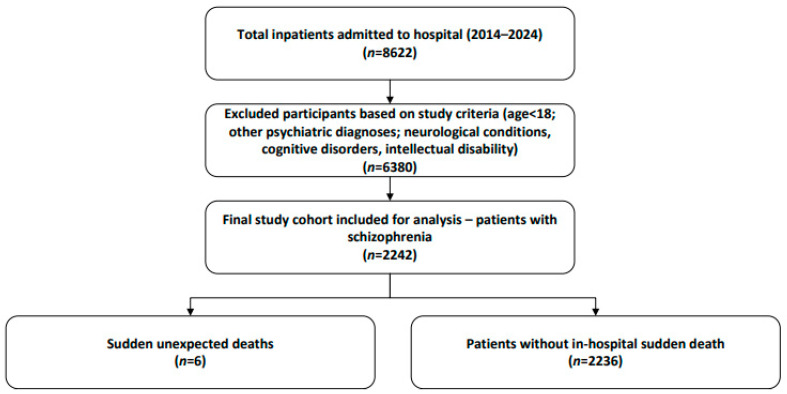
Flowchart of schizophrenia inpatients and sudden death case identification (2014–2024).

A total of 8622 patients were admitted to the Clinical Hospital of Psychiatry and Neurology, Brașov. Of these, 2242 patients (26%) had a primary diagnosis of schizophrenia. Within this subgroup, six patients died suddenly and unexpectedly during hospitalization. The process of case identification is summarized in Figure 1. In our hospital, no cases of sudden death were observed among patients with bipolar disorder, schizoaffective disorder, or major depressive disorder during the study period.

**Figure 2:**
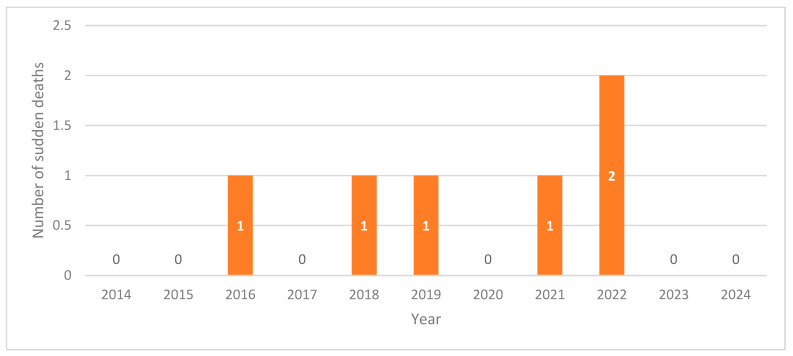
Annual distribution of sudden deaths among schizophrenia inpatients (2014–2024).

The incidence of sudden unexpected death in schizophrenia inpatients during the study period was 0.27%. Sudden deaths were distributed unevenly across the study period, with one case each in 2016, 2018, 2019, and 2021, and two cases in 2022 (Figure 2).

The study group included four men and two women. The mean age at death was 53.2 years (SD = ±17.8). The mean age of illness onset was 24.5 years (SD = ±3.3), with a mean duration of untreated psychosis (DUP) of 1.5 years (SD = ±0.6). Patients had lived with schizophrenia for an average of 28.7 years (SD = ±17.7) prior to death.

The average length of stay before death was 9.2 days (SD = 5.6). No deaths occurred within the first 24 h of admission, and only one occurred within 48 h. The remaining cases occurred later during hospitalization.

All patients were prescribed second-generation antipsychotics (SGAs) in the 24 h preceding death. The treatment distribution was as follows: paliperidone (*n* = 1.6 mg/day), quetiapine (*n* = 1.400 mg/day), clozapine (*n* = 1.600 mg/day), risperidone (*n* = 2.2–4 mg/day), and amisulpride (*n* = 1.200 mg/day). In addition, three patients were prescribed diazepam, and three received valproate in combination with antipsychotics.

### 3.1. Causes of Death

Autopsies were performed in all cases in accordance with Romanian legislation, providing confirmation of sudden death and enabling accurate classification of cardiovascular, respiratory, and asphyxial causes. Of the six sudden deaths, two were due to mechanical asphyxia, both caused by food bolus airway obstruction. Three deaths were of cardiovascular origin (pulmonary thromboembolism, myocardiosclerosis, acute myocardial infarction), while one was due to bronchopneumonia. The distribution of causes of death is summarized in Table 1.

### 3.2. Case Descriptions

Patient 1: A 30-year-old man with an 8-year history of schizophrenia died of mechanical asphyxia. Autopsy revealed obstruction of the upper airway by a food bolus. At the time of death, he was treated with risperidone 4 mg/day, diazepam 20 mg/day, and valproate 1000 mg/day. No major somatic comorbidities were documented. There were no signs of sedation that could explain the event, and it was considered an unfortunate accident (the patient was eating a cream cake while lying on the bed).

Patient 2: A 51-year-old man with long-standing schizophrenia and comorbid type 2 diabetes mellitus died of mechanical asphyxia, also related to food bolus obstruction. He was receiving clozapine 600 mg/day and diazepam 10 mg/day. Additionally, no signs of oversedation were noted, and the incident was interpreted as accidental, occurring while the patient was eating in bed.

Patient 3: A 75-year-old woman with schizophrenia and multiple cardiovascular comorbidities, including hypertension, ischemic coronary artery disease, and chronic venous insufficiency, died of pulmonary thromboembolism secondary to deep vein thrombosis. She had been receiving quetiapine 400 mg/day.

Patient 4: A 65-year-old woman with schizophrenia, atrial fibrillation, chronic heart failure (NYHA II), ischemic coronary artery disease, and a history of radiation-treated cervical neoplasm died of bronchopneumonia. At the time of death, she was treated with amisulpride 200 mg/day.

Patient 5: A 63-year-old man with schizophrenia, hypertension, and chronic venous insufficiency died of myocardiosclerosis. His treatment regimen included risperidone 2 mg/day, diazepam 20 mg/day, and valproate 900 mg/day.

Patient 6: A 35-year-old man with schizophrenia and a history of tracheostomy following removal of a subglottic foreign body one month earlier died of acute myocardial infarction. He was treated with paliperidone 6 mg/day.

### 3.3. Comparison with Historical Cohort

**Figure 3:**
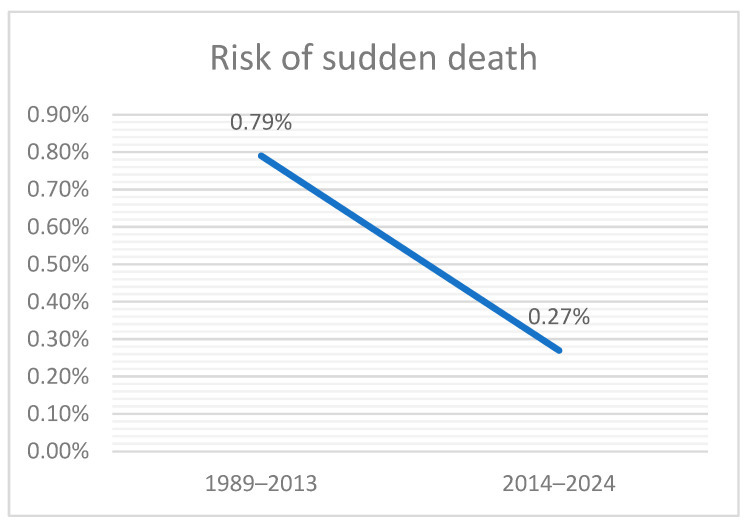
Temporal decline in the risk of sudden unexpected death among schizophrenia inpatients (1989–2024).

During the 1989–2013 period, Ifteni et al. reported 57 cases of sudden unexpected death among 7189 schizophrenia inpatients, corresponding to a risk of 0.79% (95% CI 0.61–1.03%). In the present 2014–2024 cohort, 6 sudden deaths were identified among 2242 schizophrenia inpatients, corresponding to a risk of 0.27% (95% CI 0.12–0.60%). The absolute risk difference was −0.52%, indicating a decline in incidence over time (Figure 3).

The relative risk of sudden death in the current cohort compared with the earlier one was 0.34 (95% CI 0.15–0.78), indicating a statistically significant two-thirds reduction in risk. The odds ratio likewise suggested a lower risk in the more recent cohort (OR 2.98, 95% CI 1.28–6.92, reference = earlier cohort). Both the chi-square test (χ^2^ = 6.34, *p* = 0.012) and Fisher’s exact test (*p* = 0.0068) confirmed that the decline was statistically significant.

Taken together, these analyses demonstrate a significant reduction in sudden death risk among schizophrenia inpatients in the past decade compared with the previous two decades, underscoring improvements in inpatient safety and medical care (Table 2).

An additional analysis was performed to compare the clinical and demographic characteristics of patients who died suddenly in the two cohorts. Detailed information was available for the 51 autopsied cases from the 1989–2013 cohort [6] and for all six cases identified in the 2014–2024 cohort. The comparison was therefore restricted to these autopsy-confirmed cases to maintain data uniformity and diagnostic accuracy (Table 3). There were no statistically significant differences between cohorts in mean age, sex distribution, duration of illness, or length of hospital stay. However, treatment characteristics differed substantially: first-generation antipsychotic use decreased from 83.4% to 0%, while second-generation antipsychotic use increased from 15.7% to 100% (both *p* < 0.0001). Benzodiazepine and mood stabilizer co-treatment rates did not differ significantly.

In summary, the incidence of sudden unexpected death among schizophrenia inpatients was lower during 2014–2024 than during 1989–2013. Demographic and clinical characteristics of the patients who died were similar across study periods, while differences were observed in antipsychotic treatment patterns.

## 4. Discussion

This study presents new data on sudden unexpected death among schizophrenia inpatients over the last decade (2014–2024), providing an updated assessment of its incidence, causes, and potential changes compared with earlier findings from the same institution. By directly comparing the 2014–2024 cohort with the previously reported 1989–2013 cohort, this work offers a valuable longitudinal perspective on trends in sudden death in this vulnerable population.

Our findings revealed a lower sudden death incidence in recent years and must be viewed in the context of contemporary literature on schizophrenia mortality. Most recent studies continue to highlight the elevated risk of sudden cardiac death (SCD) and premature mortality in schizophrenia. For example, a nationwide Danish analysis [9] reported an adjusted hazard ratio of 4.51 (95% CI 3.95–5.16) for SCD among people with schizophrenic disorders compared with the general population, underscoring markedly elevated risk across ages. In Lithuania (2001–2020), a cohort of 7883 individuals with schizophrenia showed all-cause standardized mortality ratio (SMR) of 1.96 (95% CI 1.88–2.04) and circulatory-disease SMR 2.17 (95% CI 2.05–2.30), with the authors concluding that people with schizophrenia have not shared in the mortality improvements seen in the general population and calling for systematic cardiovascular screening and other preventive measures [42]. Complementing this, a Romanian 10-year cohort observed 19.37% mortality (21.3 per 1000 person-years; SMR 1.58), with non-violent causes predominating—notably cardiovascular disease (27.6%) and infections (17.1%) [43]. Together, these contemporary data confirm that the mortality gap remains large.

Against this backdrop, the decline in sudden inpatient deaths in our hospital is noteworthy. It is consistent with evidence that modern treatment strategies and care models can positively influence survival in schizophrenia.

Over time, a consistent downward trend in sudden deaths has been observed. Ifteni et al. reported 22 sudden deaths among schizophrenia inpatients in the last decade of their 1989–2013 study, whereas only six such cases were identified during 2014–2024—approximately a threefold reduction. In the most recent decade, only a small number of sudden deaths occurred among schizophrenia inpatients, and none were observed among patients with bipolar disorder, schizoaffective disorder, or major depressive disorder.

The comparative analysis of autopsy-confirmed cases from both study periods further supports these observations. Because detailed clinical information was available only for the 51 autopsied patients in the 1989–2013 cohort, the comparison was limited to these cases and the six autopsied patients from the 2014–2024 cohort to ensure data completeness and diagnostic consistency. The demographic and clinical profiles of the deceased individuals—including age, sex distribution, illness duration, and length of hospitalization—were broadly similar between cohorts, indicating that patient characteristics remained stable over time. In contrast, a marked shift in treatment patterns was observed, with a complete transition from predominantly first-generation antipsychotic use to exclusive use of second-generation agents in the more recent cohort. This change mirrors broader trends in psychiatric pharmacotherapy and reflects evolving prescribing practices aimed at improving safety and tolerability.

A large nationwide cohort study reported that patients receiving first-generation oral antipsychotics had the highest mortality, whereas those treated with second-generation agents or long-acting injectables (LAIs) had significantly lower risks of death (adjusted hazard ratio for LAIs vs. oral first-generation agents ≈ 0.62) [44]. This aligns with our own findings: in the 1989–2013 report by Ifteni et al., 43 of the 51 autopsy-confirmed cases (84%) were receiving first-generation antipsychotics (FGAs), while only 8 patients (16%) were treated with second-generation agents (SGAs) at the time of death. In contrast, in our 2014–2024 cohort, all six patients (100%) were receiving SGAs during the 24 h preceding death. This marked change reflects a broader evolution in clinical practice, with less reliance on FGAs and intramuscular polypharmacy, which were frequently used in the past for severely agitated or treatment-resistant patients. Concerns about QT prolongation, torsades de pointes [45], and other arrhythmogenic effects have led to more cautious dosing and greater preference for SGAs and monotherapy when possible [46,47,48].

However, as this study is retrospective and lacks randomization, these observations should be interpreted as correlational rather than causal. The decline in sudden deaths coinciding with the wider use of SGAs may reflect a combination of improved prescribing practices, medical monitoring, and patient selection rather than a direct pharmacological effect. Moreover, while SGAs generally pose a lower cardiac risk than FGAs, they are not devoid of serious adverse effects.

The study period overlapped with the COVID-19 pandemic, during which patients with schizophrenia were shown to have increased susceptibility to infection and higher COVID-19–related mortality [49,50,51]. In our institution, the pattern of sudden inpatient deaths remained stable during this time: no cases in 2020, one in 2021, and two in 2022 (one due to mechanical asphyxia and one due to acute myocardial infarction). Importantly, none of these patients tested positive for COVID-19, as all inpatients were systematically screened at admission, on day 5, and whenever symptoms occurred. Because our hospital served as a COVID-19 support centre, psychiatric admissions during this period were largely restricted to emergencies, leading to a decrease in total inpatient volume.

Beyond COVID-19, pneumonia remains an important cause of morbidity and mortality in schizophrenia. Recent studies show that antipsychotics with high anticholinergic burden, such as clozapine, quetiapine, and olanzapine, are associated with elevated pneumonia risk [52,53]. Additional work highlights sedation, dysphagia, and medical comorbidities as significant risk factors for hospital-acquired pneumonia [54]. These observations reinforce the importance of vigilant medical monitoring and proactive management of somatic health needs in psychiatric inpatients—measures that were substantially strengthened in our hospital after 2014.

This favorable trend also reflects advances in medical monitoring and somatic care. The earlier 1989–2013 analysis emphasized that unrecognized coronary artery disease was a contributor to sudden death, concluding that “early recognition and treatment of coronary artery disease must become a clinical priority” in schizophrenia care. It appears this message has been heeded: today, there is greater collaboration with general medicine (e.g., routine internal medicine consultations and cardiology input for high-risk patients), facilitating early management of cardiovascular risk factors and conditions. The effect is likely a reduction in fatal cardiac events.

Over the past 35 years, psychiatric care has undergone a major structural transformation, including the development of community-based services and shorter hospital stays [55]. These changes may have influenced the composition of inpatient populations. However, the modern psychiatric inpatient typically represents a person with more chronic, treatment-resistant, or residually symptomatic schizophrenia, often unable to maintain independent functioning and presenting with multiple somatic comorbidities, while individuals with milder illness are increasingly managed in community settings. Such patients commonly exhibit poor self-care, limited adherence to medical therapy, and a high prevalence of cardiovascular and metabolic disorders. The most substantial improvement has been the evolution toward a multidisciplinary model of care: psychiatric inpatients are now systematically evaluated from both psychiatric and medical perspectives, with routine consultations in internal medicine, cardiology, and infectious diseases. This integrated approach facilitates early identification and management of physical illnesses that might previously have gone unrecognized. Taken together, these developments suggest that the observed decline in sudden unexpected deaths reflects advances in medical monitoring and somatic care integration rather than differences in patient selection or hospitalization criteria.

This improvement must also be considered in light of the well-documented vulnerability of patients with schizophrenia to cardiovascular disease, metabolic disturbances, and infectious illnesses [56,57]. They are also more likely to experience underdiagnosis and undertreatment of physical health conditions due to diagnostic overshadowing and fragmented care [58]. Additionally, immunological alterations and lifestyle factors (e.g., smoking, poor diet, sedentary behavior) [59] increase susceptibility to infections such as tuberculosis, hepatitis, and pneumonia [60,61,62]. These combined vulnerabilities created a strong rationale for implementing more systematic medical surveillance in psychiatric settings. Building on this, several hospital-wide measures were implemented after 2014, including routine ECGs at admission and as clinically indicated, prolactin and metabolic monitoring, echocardiographic assessment for clozapine-treated patients, and brain CT and neurological assessment. Smoking was prohibited in the hospital between 2020 and 2024, which likely contributed to lower cardiovascular risk. Routine screening for tuberculosis, lung cancer, hepatitis, and HIV has been implemented, along with tighter control of antibiotic use and regular access to infectious disease consultations when needed. Both pre-existing infections at admission and nosocomial infections, although rare in our hospital, are managed by an infectious disease specialist, and patients are regularly screened for MRSA and other pathogens. At admission, all patients undergo systematic screening for life-threatening medical conditions. Together, these interventions represent a significant modernization of inpatient care and likely contributed to the downward trend observed.

Clinically, the findings of this study underline the importance of approaching patients with schizophrenia as whole individuals whose psychiatric symptoms cannot be separated from their physical health. Comprehensive somatic assessment should accompany every psychiatric evaluation, with particular attention to cardiovascular, metabolic, and respiratory comorbidities. Routine investigations—including ECG, metabolic panels, and targeted imaging or specialist consultations when indicated—can aid in the early detection of life-threatening conditions that may otherwise remain unrecognized. Regular interdisciplinary collaboration between psychiatry, internal medicine, cardiology, and other specialties is essential to ensure timely diagnosis and management of medical complications. This integrated model of care promotes both psychiatric stability and physical health, representing the most effective strategy for reducing preventable mortality in this vulnerable population.

Finally, we must consider the impact of targeted interventions to reduce airway-related deaths, such as aspiration and choking. The two deaths due to mechanical asphyxia observed in this study emphasize that, despite substantial advances in medical and psychiatric care, airway-related incidents continue to pose a serious safety concern in inpatient settings. Historically, such events accounted for approximately 7.8% of sudden deaths in our hospital [6], reflecting the persistent vulnerability of patients with schizophrenia to dysphagia and aspiration. These events reflect well-recognized risk factors in this population, combining medication-related influences—such as sedation, anticholinergic effects, and extrapyramidal symptoms—with patient-related vulnerabilities including edentulism, advanced age, and disregard for safety measures, such as eating in bed or consuming unsuitable foods [63]. Recognizing the elevated risk of aspiration and choking in psychiatric inpatients, our institution implemented structured dysphagia risk screening and modified feeding protocols. Patients receiving clozapine or with a prior history of choking are routinely evaluated for swallowing difficulties, and individualized dietary modifications are made to minimize aspiration risk. Sedated or cognitively impaired patients are given additional supervision during meals. Despite these measures, mechanical asphyxia accounted for 2 of the 6 deaths in our cohort, indicating that this complication persists as an important concern. This risk factor should always be considered in the management of schizophrenia inpatients. Airway safety should remain a central focus of inpatient care, with continued monitoring and refinement of protocols to further reduce preventable fatalities.

## 5. Strengths and Limitations

This study has several strengths. First, mandatory autopsy for all inpatient deaths ensured accurate cause-of-death classification, avoiding the misclassification common in studies relying solely on death certificates. Second, the single-centre design with a consistent catchment area and autopsy practice from 1989 to 2024 allowed a meaningful assessment of temporal changes without inter-institutional variability. Nonetheless, certain limitations should be acknowledged. The retrospective design relies on existing records. The number of events was small in the 2014–2024 cohort, limiting subgroup analyses and producing relatively wide confidence intervals (RR 0.34, 95% CI 0.15–0.78). Detailed demographic data for the entire 1989–2013 inpatient cohort were unavailable; therefore, the comparative analysis was limited to autopsy-confirmed cases to ensure data consistency across study periods. In addition, the demographic and clinical profile of admitted patients may have changed over time—for example, the current inpatient population may include fewer severely medically ill individuals, and more cases may be managed in outpatient settings—all of which could partially explain the observed decline. Finally, generalizability is limited by the single-hospital setting and Romania-specific practices. Even with complete autopsy examination, certain underlying mechanisms—such as subclinical arrhythmogenic, metabolic, or molecular abnormalities—may remain undetected using conventional pathological techniques. Future studies should therefore aim to integrate multicentre datasets, advanced molecular or genomic analyses, and prospective physiological monitoring to better characterize the biological pathways of sudden death in schizophrenia and to inform targeted prevention strategies.

## 6. Conclusions

This long-term comparative study provides updated evidence on sudden unexpected death among schizophrenia inpatients over a 35-year period. The incidence of sudden death was lower during 2014–2024 compared with 1989–2013, suggesting that advances in clinical practice, stronger collaboration between psychiatry and general medicine, and the use of antipsychotics with improved safety profiles have contributed to greater inpatient safety. Although the demographic and clinical characteristics of affected patients were comparable across study periods, treatment patterns evolved, with second-generation agents replacing earlier first-generation regimens.

These findings indicate that systematic medical monitoring and regular cardiovascular assessment can substantially reduce preventable deaths among psychiatric inpatients. Sustained implementation of integrated care models—combining psychiatric treatment with proactive management of physical health—remains essential to preserve and extend these gains.

A few cases of sudden death still occurred in the recent decade, indicating that this risk cannot be completely eliminated even under improved clinical conditions. Continued vigilance, individualized risk evaluation, and close cooperation between psychiatry and general medicine are important for maintaining patient safety. Regular review of clinical outcomes will help ensure that the progress achieved in reducing mortality among patients with schizophrenia is maintained over time.

## Figures and Tables

**Table 1:** Autopsy-confirmed causes of sudden death in schizophrenia inpatients.

Cause of Death	Number of Patients (*n* = 6)
Mechanical asphyxia	2 (33.33%)
Cardiovascular causes	3 (50%)
Respiratory infection (bronchopneumonia)	1 (16.67%)

**Table 2:** Comparison of sudden deaths in schizophrenia inpatients (1989–2013 vs. 2014–2024).

Study Period	Total Schizophrenia Patients	Sudden Deaths	Deaths per 1000 Inpatients	Annualized Deaths
1989–2013	7189	57	7.93	2.375
2014–2024	2242	6	2.68	0.60

**Table 3:** Comparative demographic and pharmacological profile of schizophrenia patients with sudden unexpected death across two study periods.

Characteristics	1989–2013 [6] (*n* = 51)	2014–2024 (*n* = 6)	*p*-Value
Age (years ± SD)	55.9 ± 9.4	53.2 ± 17.8	0.55
Male gender (%)	56.9	66.7	0.65
Duration of schizophrenia (years ± SD)	27.7 ± 10.3	28.7 ± 17.7	0.83
Length of stay (days ± SD)	11.7 ± 7.6	9.2 ± 5.6	0.4356
First-generation antipsychotics (%)	83.4	0	<0.0001
Second-generation antipsychotics (%)	15.7	100	<0.0001
Benzodiazepines (%)	74.5	50	0.21
Mood stabilizers (%)	35.3	50	0.48

Data for 1989–2013 derived from Ifteni et al. [6]. Comparison restricted to autopsy-confirmed cases to ensure diagnostic consistency between cohorts.

## Data Availability

The data supporting the findings of this study are available from the corresponding author upon reasonable request.

## Abbreviations

TNF-α	Tumor Necrosis Factor Alpha
IL-6	Interleukin 6
PLN	Phospholamban
ANKRD1	Ankyrin Repeat Domain 1
CALN1	Calneuron 1
CACNA1A	Calcium Voltage-Gated Channel Subunit Alpha1 A
RNA	Ribonucleic Acid
VCAM-1	Vascular Cell Adhesion Molecule 1
ICAM-1	Intercellular Adhesion Molecule 1
VEGF	Vascular Endothelial Growth Factor
LAI	Long-Acting Injectable
SCD	Sudden cardiac death
SMR	Standardized mortality ratio
DUP	Duration of untreated psychosis
SD	Standard deviation
FGAs	First-generation antipsychotics
SGAs	Second-generation antipsychotics
